# Relationship between the image characteristics of artificial intelligence and EGFR gene mutation in lung adenocarcinoma

**DOI:** 10.3389/fgene.2022.1090180

**Published:** 2023-01-04

**Authors:** Guoping Zhou, Shuhua Xu, Xiaoli Liu, Jingjun Ge, Qiyu He, Weikang Cao, Junning Ding, Xinghua Kai

**Affiliations:** ^1^ Department of Cardiothoracic Surgery, Dongtai Hospital of Traditional Chinese Medicine, Dongtai, China; ^2^ Department of Pathology, Dongtai Hospital of Traditional Chinese Medicine, Dongtai, China; ^3^ Department of Radiology Imaging, Dongtai Hospital of Traditional Chinese Medicine, Dongtai, China

**Keywords:** lung adenocarcinoma, EGFR gene mutations, artificial intelligence, intelligent medicine, image characteristics

## Abstract

Lung Adenocarcinoma (LUAD) is a kind of Lung Cancer (LCA) with high incidence rate, which is very harmful to human body. It is hidden in the human body and is not easy to be discovered, so it brings great inconvenience to the treatment of LUAD. Artificial Intelligence (AI) technology provides technical support for the diagnosis and treatment of LUAD and has great application space in intelligent medicine. In this paper, 164 patients with primary LUAD who underwent surgery in Hospital A from January 2020 to December 2021 were selected as the study subjects, and the correlation between the imaging characteristics of LUAD and Epidermal Growth Factor Receptor (EGFR) gene mutation was analyzed. Finally, the conclusion was drawn. In terms of the study on the correlation between EGFR mutation of LUAD and the imaging characteristics of Computed Tomography (CT), it was concluded that there were significant differences between the patient’s sex, smoking history, pulmonary nodule morphology and the EGFR gene, and there was no significant difference between the patient’s tumor size and EGFR gene; in the study of the relationship between EGFR gene mutation and CT signs of LUAD lesions, it was found that there were significant differences between the symptoms of cavity sign, hair prick sign and chest depression sign and EGFR gene, but there was no significant difference between the symptoms of lobulation sign and EGFR gene; in the study of pathological subtype and EGFR gene mutation status of LUAD patients, it was concluded that the pathological subtype was mainly micropapillary. The mutation rate was 44.44%, which was the highest; in terms of CT manifestations of adjacent structures of lung cancer and the study of EGFR gene mutation status, it was found that there was a statistical difference between the tumor with vascular convergence sign and EGFR gene mutation, and pleural effusion, pericardial effusion, pleural thickening and other signs in tumor imaging were not significantly associated with EGFR gene mutation; in terms of the study of CT manifestations of adjacent structures of LCA and EGFR gene mutation status, it was concluded that pleural effusion, pericardial effusion, pleural thickening and other signs in tumor images were not significantly associated with EGFR gene mutation; in terms of analysis and cure of LUAD, it was concluded that the cure rate of patients was relatively high, and only a few people died of ineffective treatment. This paper provided a reference for the field of intelligent medicine and physical health.

## 1 Introduction

The imaging features of LUAD play an important role in the treatment and research of LCA, and the research on them can provide reference for intelligent medical treatment and cure of LUAD. With the rapid development of artificial intelligence technology, intelligent medical treatment has brought great convenience to the treatment of LUAD. It has become a general trend to analyze the correlation between the image characteristics of LUAD and EGFR gene mutation. At the same time, due to the huge and complex data set of lung cancer gene expression profile, it is necessary to extract effective information through artificial intelligence methods.

The imaging features of LUAD have been deeply studied in the field of intelligent medicine. Yu Lingming used a mountain learning arrangement based on CT image characterization to predict the pathological staging of non-small cell LCA (Yu et al., 2019). Yoon Jiyoung predicted the effect of programmed acellular death ligand one presentation on advanced LUAD by CT imaging (Yoon et al., 2020). Koyasu Sho concluded that gradient tree boosting was useful for forecasting the pathologic subtypes of non-small cell LCA and the polyester/18F pentafluoromethacrylate interferon for EGFR mutation status was useful (Koyasu et al., 2020). Pascoe Heather M analyzed the multifaceted nature of LUAD (Pascoe et al., 2018). She Yunlang investigated the predictable worth of CT-based radiology in distinguishing inert LUAD from aggressive LUAD in patients with lung nodules (She et al., 2018). Gertych Arkadiusz found that convolutional neural networks could discriminate accurately between the four tissue growth patterns of LUAD in digital slides (Gertych et al., 2019). Abdul Jabbar Khalid believed that geographic immune variability illuminated the differential evolution of LUAD (AbdulJabbar et al., 2020). There are many researches on LUAD, but there is no research on artificial intelligence in this field.

EGFR gene mutation has a certain correlation with cancer treatment, and many scholars have also made achievements in this field. Li Yajun analyzed the effect of CT slide depth and convoluted kernels on the performance of radiological models for forecasting EGFR statuses in non-small cell LCA (Li et al., 2018). Jia Tian-Ying modeled by using the characteristics of radiology and random forest, and identified the EGFR mutation of LUAD through non-invasive imaging (Jia et al., 2019). Mei Dongdong analyzed CT images of lung adenocarcinoma and explored whether cell characteristics could become a substitute biomarker for EGFR mutation (Mei et al., 2018). Tulchinsky Eugene analysed the escape mechanism of EGFR-targeted treatment in LCA (Tulchinsky et al., 2019). Kazue Yoneda studied the treatment process and protocols for non-small cell LCA with EGFR deletions (Yoneda et al., 2019). Cicek Tugba analysed the adequacy of Endotracheal Ultrasonography-tobronchial Needle Aspiration (EBUS-TBNA) specimens for mutation analysis in LCA (Cicek et al., 2019). Vyse Simon elucidated the structure of the crystal structure of mutant kinases with EGFR exon 20 insertion and revealing a unique mechanism of kinase initiation and spatial configuration, which determined the lack of reaction of these EGFR synapses to commercially approved EGFR injectants (Vyse and Huang, 2019). Although there are many studies on EGFR and LCA, the research in this area is not deep enough.

This study retrospectively analyzed 164 patients with primary LUAD who underwent surgery in Hospital A from January 2020 to December 2021. The EGFR gene detection results of these patients are available, which could improve the research speed of LUAD and accelerate its progress. The purpose of this study is to explore the relationship between these patients and general clinical features in order to better guide clinical management.

## 2 Feature extraction of LUAD image based on artificial intelligence

### 2.1 Artificial intelligence and medical health

The wide application of medical imaging is mainly driven by the progress of computer vision technology. However, there is a serious shortage of imaging and radiotherapy doctors, and there is a shortage of doctors with rich and high-quality clinical experience. Due to the vision and experience evaluation of imaging doctors, there are many cases of misdiagnosis (Bianconi et al., 2019; Wang et al., 2019). The speed of medical imaging operators reading films and the speed of radiation therapists drawing target areas are time-consuming. In medical imaging, the use of artificial intelligence can help doctors read films and draw targets, which would save doctors a lot of time and improve the accuracy of diagnosis, radiotherapy and surgery. With the support of artificial intelligence technology, feature extraction of lung adenocarcinoma image has more possibilities.

### 2.2 Feature recognition algorithm of LUAD image based on artificial intelligence

**FIGURE 1:**
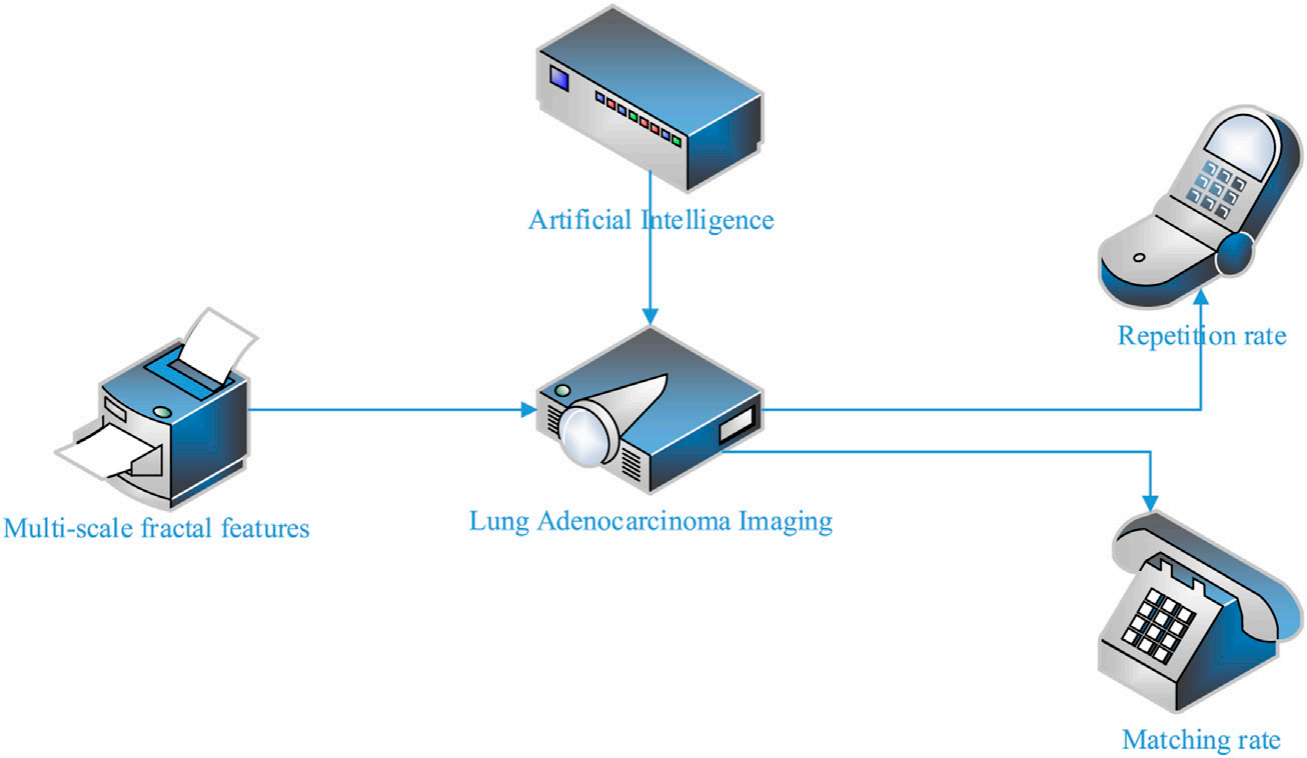
AI-based LUAD image feature recognition algorithm.

The design process of feature recognition algorithm for LUAD image based on artificial intelligence is recorded in Figure 1.

The multi-scale fractal feature is a fractal parameter change measurement function (Wu et al., 2020). The multi-scale fractal feature can be understood as the change degree of D dimension area $\left(K\left(x,y,\varepsilon \right)\right)$ within the scale range of $\varepsilon_{\max}$ . The multi-scale fractal feature is used to highlight the difference in fractal features between man-made targets and natural background

$$MFFK\left(x,y\right)={\overset{\varepsilon_{\max}}{\underset{\varepsilon =2}{\sum }}\left[K\left(x,y,\varepsilon \right)-\frac{1}{\varepsilon_{\max}-1}\overset{\varepsilon_{\max}}{\underset{\varepsilon =2}{\sum }}K\left(x,y,\varepsilon \right)\right]}^{2}$$ (1)

The threshold value is set to 5, and the binary LUAD image is obtained by segmentation of the enhanced LUAD image as follows:

$$\theta =\frac{\tan^{-1}\left(\frac{2\left(\frac{M_{11}}{M_{00}}-x_{c}y_{c}\right)}{\left(\frac{M_{20}}{M_{00}}-x_{c}^{2}\right)-\left(\frac{M_{02}}{M_{00}}-y_{c}^{2}\right)}\right)}{2}$$ (2)

In the formula, M_00_ is the zero matrix, and $\left(x_{c},y_{c}\right)$ represents the centroid of LUAD image.

The n-order m-fold Zernike moment of a discrete image is defined as follows:

$$Z_{n,m}=\frac{\left(n+1\right)}{\pi {\left(N-1\right)}^{2}}\overset{N-1}{\underset{x=0}{\sum }}\overset{N-1}{\underset{y=0}{\sum }}I\left(x,y\right)R_{n,m}\left(\rho \right)e^{jim\theta }$$ (3)

In the formula, *n* = 0,1,2,...; $0\leq \left|m\right|\leq n$ ; $n-\left|m\right|$ is an even number. (p,θ) is the polar coordinate representation under the unit circle, and R_n,m_ is the radial polynomial.

In order to optimize discrete problems, this paper proposes an artificial intelligence algorithm to update them:

$$v_{ij}=\left\{\begin{array}{l} v_{ij}^{1},\\ v_{ij}^{0}, \end{array}\right.\begin{array}{l} x_{ij}=0\\ x_{ij}=1 \end{array}$$ (4)

In the formula, $v_{ij}^{1}$ and $v_{ij}^{0}$ respectively represent the probability that the *j*th position of the *i*th particle becomes one or 0.

The calculation method of (Eq. 4) still has some defects, so this paper improves the particle position. The update formulas of particle position are as follows:

$$x_{ij}\left(t+1\right)=\left\{\begin{array}{l} {\bar{x}}_{ij}\left(t\right),\\ x_{ij}\left(t\right), \end{array}\right.\begin{array}{l} r_{ij}\leq v_{ij}^{\prime }\\ r_{ij}>v_{ij}^{\prime } \end{array}$$ (5)

$$v_{ij}^{\prime }\left(t\right)=sig\left(v_{ij}\left(t\right)\right)$$ (6)

In the formula, ${\bar{x}}_{ij}\left(t\right)$ represents the inversion of $x_{ij}\left(t\right)$ in binary, and r_ij_ is a random number in the $\left[0,1\right]$ interval.

The repetition rate is the ratio of the number of repeated feature points extracted from two similar images to the total number of extracted points (Gong et al., 2020). The higher the repetition rate of feature points, the more stable the extracted feature points are and the higher the correct matching rate of feature points would be.

The formula for calculating the repetition rate is as follows:

$$R=\frac{N_{3}}{\min\left(N_{1},N_{3}\right)}\times t$$ (7)

In the formula, R is the repetition rate; N1 and N2 are the number of feature points extracted from the left and right images respectively; N3 is the number of feature points from the left image projection to the right image; t is the overlap of similar images.

The ratio k between the number of matches obtained from fine matching of two images and the average value of feature points extracted from the two images is the image matching rate.

The matching rate is calculated as follows:

$$k=\frac{N}{N_{1}+N_{2}}\times t$$ (8)

In the formula, N_1_ and N_2_ are the feature points extracted from the two images; t is the overlap of similar images.

## 3 Intelligent medical and biological information

With the development of technology and the aging of the population, AI would become a part of many industries in the future, including medical technology (Ge et al., 2019). Although computers would never replace humans, they are useful for certain tasks and can improve the patient’s experience. However, the benefits are much greater. The combination of artificial intelligence and medical technology would simplify medical care and create new opportunities. In the case of declining profits and increasing government regulations, it saves money and promotes the survival and development of pharmaceutical enterprises. AI also provides the possibility to standardize many processes in the field of healthcare. Just as the specific procedures are different, many policies and practices are the same. Patients often do not understand their rights and responsibilities in health and safety and the accuracy of their data.

**FIGURE 2:**
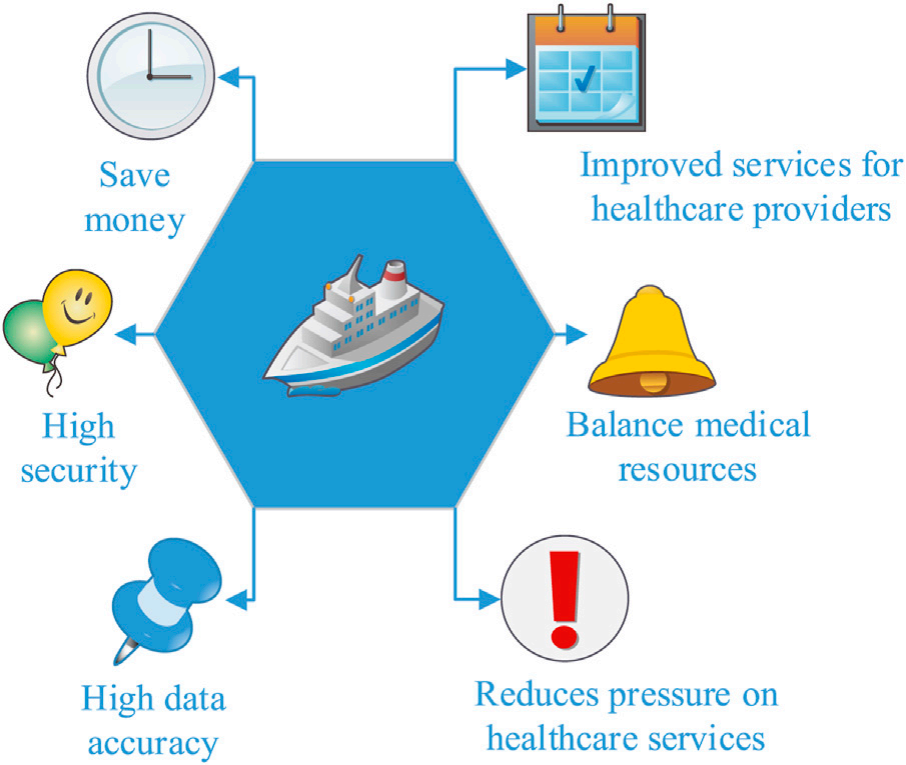
Advantages of smart healthcare.

The development of AI medical platform would help medical institutions improve services and balance medical resources, thereby reducing the pressure on medical services, especially in areas with limited medical resources (Zhang et al., 2020). Medical institutions choose different construction methods to improve their medical services according to different information technology levels. The AI platform consists of data, computing power, open source systems and algorithms, and various technologies. The computing power ensures the speed of the AI platform. The advantages of AI in medical care are recorded in Figure 2.

In the field of data driven auxiliary medical diagnostic imaging, applications include brain tumor segmentation, pulmonary nodule detection, Alzheimer’s disease detection, lymph node detection, pulmonary bronchiectasis detection, chest disease detection and liver ultrasound detection. Each patient has an average of 20–30 images, such as the medical imaging of pulmonary nodules. Computer vision models, such as residual neural networks, are usually used to automatically identify pulmonary nodules. It can train neural networks with dozens or even hundreds of layers, which requires high computing power. The huge amount of data increases the computing time. Therefore, the development of a supercomputing platform can not only reduce computing time, but also improve medical efficiency and reduce patient waiting time.

Knowledge based design creates high value medical diagnostic cards. The knowledge map is a typical product of the big data era. The combination of big semantic network, big data technology and deep learning technology is becoming the main driving force for the development of artificial intelligence. The medical knowledge map mainly uses four technologies: knowledge representation, knowledge extraction, knowledge fusion and knowledge reasoning.

Artificial intelligence has been applied to LUAD, thyroid cancer, breast nodules and other tumors, coronary artery plaque, skin cancer, liver pathology and many other fields. Early LCA is asymptomatic, but 70%–80% of patients with advanced LCA lost the opportunity of surgery and more and more of them developed into LCA. Although most pulmonary nodules are benign, the proportion of early LCA is high and benign.

## 4 Significance of imaging characteristics and EGFR gene mutation in LUAD

**FIGURE 3:**
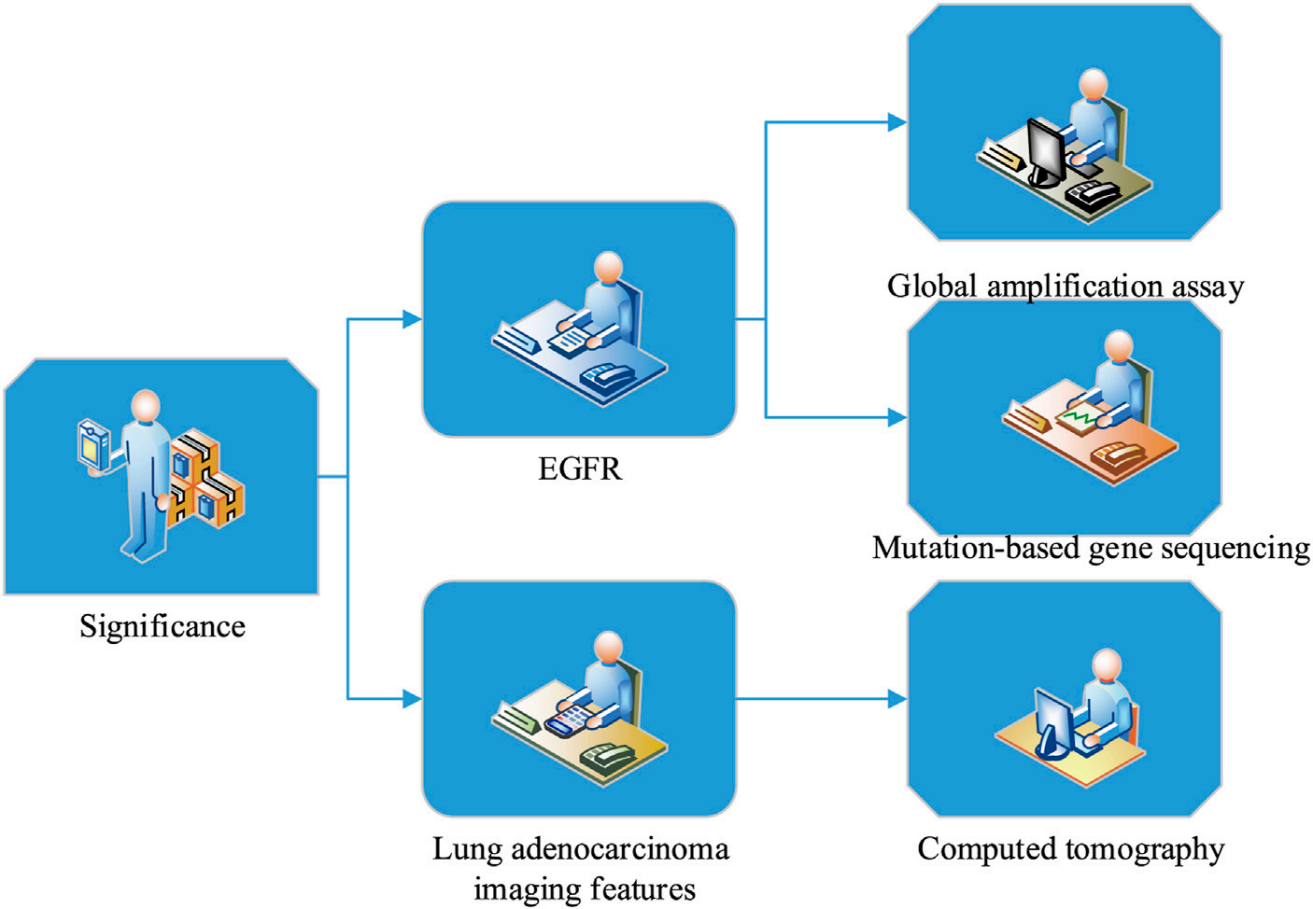
Significance of LUAD imaging features and EGFR gene mutation study.

The research significance of imaging characteristics of LUAD and EGFR gene mutation is discussed from two aspects: LUAD imaging and EGFR gene mutation, as shown in Figure 3.

Lung cancer is one of the most common cancers in the world, with the highest mortality. Lung adenocarcinoma is the most common histological type of lung cancer. Small cell LCA accounts for 92% of LCA. EGFR mutation is the main subtype of LUAD. About 45% of non-smoking Non Small Cell LCA (NSCLC) patients have EGFR mutation. Although EGFR mutations are more common in women and non-smokers, there are no reliable clinical features that can accurately distinguish EGFR phenotype from LCA. In clinical practice, EGFR phenotype and LCA are distinguished by gene detection of tissue samples. Gene detection is not only costly and time-consuming, but also invasive due to tissue sampling. Computed tomography is the most popular and widely used imaging method in LCA, because it provides excellent spatial resolution information about the microstructure of LCA, which may reflect the genetic phenotype of the tumor.

EGFR has been proved to be abnormally or highly expressed in many solid tumors and can regulate various biological activities of tumor cells, which inhibit apoptosis through its downstream signal transduction. The mutation of EGFR receptor gene may partly predict the sensitivity and efficacy of targeted therapy. At present, the mutation status of EGFR receptor is mostly determined by global amplification detection or mutation based gene sequencing, which requires expensive and complex surgery or puncture technology to obtain tissue samples. In recent years, molecular targeted therapy has become a new hot spot in the treatment of lung cancer, and EGFR gene mutation has attracted great public attention.

## 5 Correlation between imaging characteristics of LUAD and EGFR gene mutation

### 5.1 Selection of test objects

From January 2020 to December 2021, 164 patients with primary LUAD who underwent surgery in Hospital A were retrospectively analyzed and their basic information was recorded in Table 1.

**TABLE 1:** Basic information about the test subjects.

Basis of classification	Subgroup	Number
Gender	Male	86
	Female	78
Genotype	Mutant	64
	Wild type	100

From January 2020 to December 2021, the average age of 164 patients with primary LUAD who underwent surgery in Hospital A was (53.5 ± 2.2) years old. Of these patients, 86 were women and 78 were men. 64 patients were mutated and 100 patients were wild type. In general, the probability of men suffering from LUAD is higher than that of women, and the wild type patients are more than the mutant type patients.

Inclusion criteria: Patients with pulmonary nodules or masses found on standard chest CT; untreated lesions with clinical anti-cancer treatment before the study; surgical intervention was carried out after all chest CT scans, and tissue samples were collected for pathological classification and EGFR gene analysis.

Exclusion criteria: Chest CT scanning does not meet the inclusion criteria; chest CT scanning is performed in other institutions. There is no postoperative tissue available for EGFR gene detection, and the imaging data are incomplete.

### 5.2 CT measurement and film reading method

All patients were placed in supine position with arms raised, and spiral chest examination was performed with Siemens dual source CT scanner. Tumor features include tumor size, lung lobe sign, hair prick sign, pleural traction sign, air branch sign, bilateral lung metastasis and pleural effusion. The tumor size, lung lobe sign, hair prick sign, pleural traction sign, air bronchogram sign, the presence of bilateral lung metastasis and pleural effusion were recorded one by one on chest CT. During the examination, the size of the tumor is measured by the diameter of the longest lung window. If it is multiple lesions, it is measured by the diameter of the largest lesion.

The film would be read independently by three doctors above medical level. If the readings differ, the three doctors would discuss and resolve the issue. The film reading content includes: The lung lobe where the disease is located, the density of the disease, and the signs of lobation, cavitation, folding, pleural indentation and lymph node enlargement.

Lesion density: This is divided into pure ground glass density and density with solid components.

Lymph node enlargement: In a defined area, lymph nodes with a short diameter>1 cm are enlarged.

### 5.3 Correlation between imaging characteristics of LUAD and EGFR gene mutation

#### 5.3.1 Correlation between EGFR mutation and chest CT imaging features in LUAD

In order to thoroughly analyze the correlation between the imaging characteristics of LUAD and EGFR gene mutation, this paper studied the correlation between the mutation type, sex, smoking history and tumor size based on the EGFR mutation and chest CT imaging characteristics of LUAD, and recorded the results in Table 2.

**TABLE 2:** Correlation of EGFR mutations in LUAD with chest CT imaging features.

Group	Gender		Smoking history		Tumor size		Pulmonary nodule pattern	
	Male	Female	No	Yes	Pulmonary nodules≤3 cm	Pulmonary masses>3 cm	Partially solid	Completely solid
Genome group	42	56	35	29	49	15	28	36
Wild group	44	22	64	36	60	40	48	52
Total	86	78	99	65	109	55	76	88
Mutation rate/%	48.84	71.79	35.35	44.62	44.95	27.27	36.84	40.91
p	012		024		325		024	

In 164 patients, 42 were male patients with mutation type, and the mutation rate was 48.8 (42/86). 56 female patients were mutated, the mutation rate was 71.79% (56/78). There was a significance difference between the both of them (*p* = .012); 35 patients had no smoking history, and the mutation rate was 35.35% (35/99). 29 patients had smoking history, the mutation rate was 44.62% (29/65). There was a significance difference between the both of them (*p* = .024); there were 49 patients with tumors ≤ 3cm, and the mutation rate was 44.95% (49/109). 15 patients had tumors>3cm, and the mutation rate was 27.27% (15/55). There was no meaningful difference between them (*p* > .05); the number of patients with EGFR mutation type pulmonary nodules with partial solid morphology was 28, and the mutation rate was 36.84% (28/76). There were 36 patients with EGFR gene mutation whose pulmonary nodules were completely solid, and the mutation rate was 40.91% (36/88). There was a significance difference between the both of them (*p* = .024).

#### 5.3.2 The relationship between EGFR gene mutation and CT findings in LUAD

Mutation of EGFR gene in lung adenocarcinoma lesions represents the symptoms of lung adenocarcinoma, which can analyze the relationship between lung adenocarcinoma symptoms and CT signs and provide reference for correlation analysis between imaging features of lung adenocarcinoma and EGFR gene mutation. The test results of the relationship between EGFR gene mutation and CT signs of LUAD lesions are summarized in Table 3.

**TABLE 3:** Relationship between EGFR mutations and CT signs in LUAD lesions.

Group	Lobar sign		Cavity sign		Burr sign		Pleural depression sign	
	No	Yes	No	Yes	No	Yes	No	Yes
Genome group	34	30	46	18	33	31	18	46
Wild group	49	51	67	33	58	42	51	49
Total	83	81	113	51	91	73	69	95
Mutation rate/%	40.96	37.04	40.71	35.29	36.26	42.47	26.09	48.42
p	316		023		034		005	

From January 2020 to December 2021, among 164 patients with primary LUAD who underwent surgery in Hospital A, 81 patients showed lobulation sign, with a mutation rate of 37.04% (30/81). There was no meaningful difference (*p* > .05); 51 patients showed cavitation sign, and the mutation rate was 35.29% (18/51). The difference was statistically meaningful (*p* = .023); 73 patients showed spicule sign, and the mutation rate was 42.47% (31/73). The difference was statistically meaningful (*p* = .034); there were 95 patients with chest depression sign, and the mutation rate was 48.42% (46/95). The difference was statistically meaningful (*p* = .005).

#### 5.3.3 Pathological subtypes and EGFR gene mutation in patients with LUAD

**FIGURE 4:**
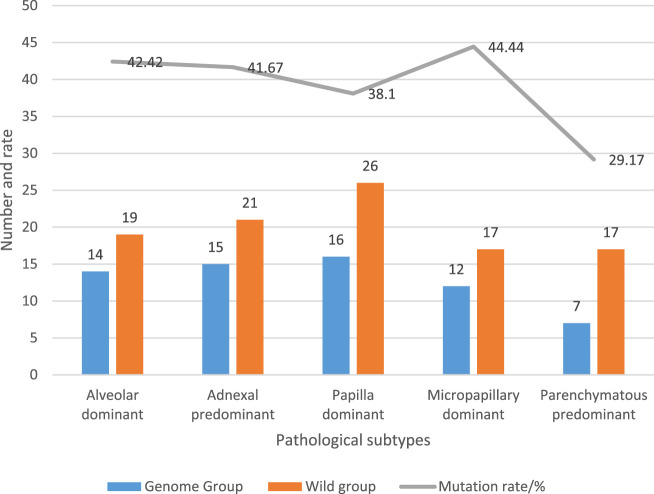
Pathological subtypes and EGFR mutation status in LUAD patients.

All cases of lung adenocarcinoma were classified into histological subtypes according to the international multidisciplinary classification of lung adenocarcinoma, including acinar dominated, adherent dominated, papillary dominated, micropapillary dominated and solid dominated. The determination results of pathological subtypes and EGFR gene mutation status of LUAD patients are recorded in Figure 4.

Among 164 patients with LUAD, 14 patients had acinar dominant pathological subtype, with a mutation rate of 42.42% (14/33); the pathological subtype of 15 patients was mainly adherent type, and the mutation rate was 41.67% (15/36); the pathological subtype of 16 patients was mainly nipple type, and the mutation rate was 38.1% (16/42); the pathological subtype of 12 patients was mainly micropapillary type, and the mutation rate was 41.38% (12/29); the pathological subtype of seven patients was mainly solid type, and the mutation rate was 29.17% (7/24).

#### 5.3.4 CT manifestations of adjacent structures of LCA and mutation status of EGFR gene

LUAD belongs to non-small cell carcinoma. The growth of LCA cells would affect the changes in the structure of surrounding cells, so it is of practical significance to investigate it. The CT findings of adjacent structures of LCA and the investigation results of EGFR gene mutation status are shown in Table 4.

**TABLE 4:** CT presentation of adjacent structures and EGFR mutation status in LCA.

Parameters	Genome group	Wild group	p
Vascular cluster sign +	21	65	035
Vascular cluster sign-	43	35	
Pleural effusion+	27	21	35
Pleural effusion-	37	79	
Pericardial effusion+	33	48	65
Pericardial effusion-	31	52	
Thoracic thickening+	31	34	64
Thoracic thickening-	33	66	

The number of mutations in the tumor group with vascular convergence sign was 21, and the number of mutations in the group without vascular convergence sign was 43. There was a statistical difference between them (*p* < 05). In addition, pleural effusion, pericardial effusion, pleural thickening and other signs in tumor images were not significantly associated with EGFR, gene mutation (*p* > 05).

#### 5.3.5 The relationship between EGFR gene mutation and clinical stage of LCA in patients with LUAD

**FIGURE 5:**
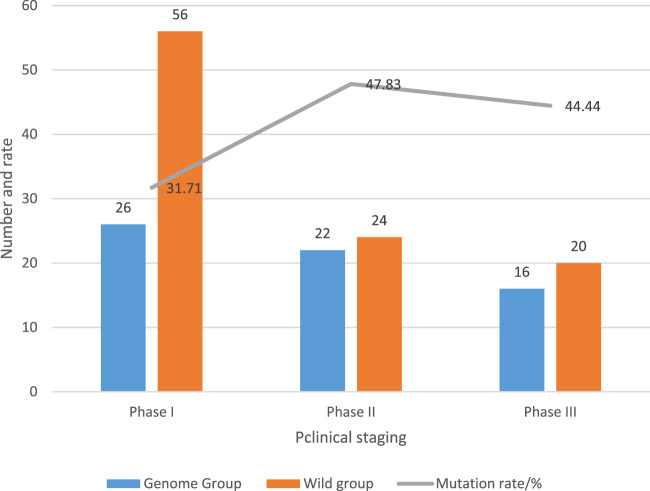
The relationship between EGFR gene mutations and clinical stages of LCA.

According to the symptoms and types of LUAD, the clinic can be divided into stage I, stage II and stage III. The relationship between EGFR gene mutation and clinical stage of LUAD patients is recorded in Figure 5.

26 patients with clinical stage Ⅰ had mutation, and the mutation rate was 31.71% (26/82); 22 patients in stage Ⅱ had mutations, and the mutation rate was 47.83% (22/46); there were 16 cases of mutation in stage III patients, and the mutation rate was 44.44% (16/36).

#### 5.3.6 Analysis and cure of LUAD

**FIGURE 6:**
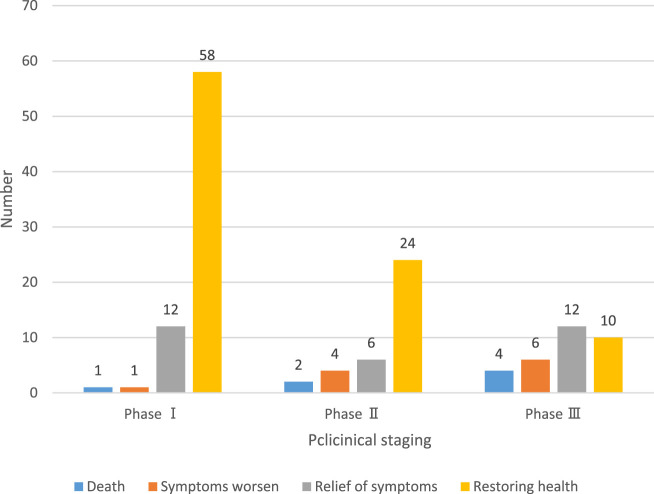
Analysis and cure of adenocarcinoma of the lung.

In order to analyze the treatment of patients, 164 patients were interviewed and investigated to explore whether the image feature analysis of pulmonary adenocarcinoma based on artificial intelligence can cure the patients’ pulmonary adenocarcinoma and promote their health. After excluding the patients who could not be contacted, the actual number of people in this survey was 140, and the physical conditions of these 140 people were recorded in Figure 6.

Among the patients in clinical stage I, 58 recovered, 12 were relieved, one was aggravated and one died; among the patients in clinical phase II, 24 recovered, six were relieved, four were aggravated and two died; among the patients in clinical phase III, 10 recovered, 12 were relieved, six were aggravated and four died. In general, the cure rate of patients is high, and only a few people died of ineffective treatment.

## 6 Conclusion

In order to analyze the correlation between image features of LUAD and EGFR gene mutation, and improve the cure rate of LUAD, this paper designed an artificial intelligence algorithm to analyze the correlation between image features of LUAD and EGFR gene mutation. It also designed a test to describe it, and finally reached a feasible conclusion. There were significant differences between the EGFR mutation rate and the sex, smoking history, pulmonary nodule morphology, patients with cavity sign, hair prick sign, thymus depression sign and tumor with vascular convergence sign groups of LUAD patients. There was no meaningful difference between tumor size, lobulated sign, pleural effusion, pericardial effusion, pleural thickening and EGFR mutation rate. According to the interview and investigation of patients, the cure rate of LUAD was greatly improved, which showed that AI could propose effective cure measures to improve the treatment plan of patients.

## Data Availability

The original contributions presented in the study are included in the article/supplementary material, further inquiries can be directed to the corresponding authors.
